# The Water Extract of Sweet Tea Alleviates LPS-Induced Acute Lung Injury Through Anti-Inflammatory and Antioxidant Effects

**DOI:** 10.3390/nu17213425

**Published:** 2025-10-31

**Authors:** Haorui Zheng, Taoyu Wang, Hairui Xue, Zihan Zhang, Hengyang Zhang, Yang Cao, Lin Tang

**Affiliations:** College of Life Sciences, Sichuan University, Chengdu 610000, China

**Keywords:** sweet tea, ALI, pyroptosis, antioxidant, anti-inflammatory

## Abstract

**Background/Objectives:** *Lithocarpus litseifolius* (Hance) Chun, also known as sweet tea, is a traditional Chinese tea-making plant. Acute lung injury (ALI), a life-threatening syndrome with symptoms like hypoxemia and dyspnea, can be triggered by infection or trauma, with high morbidity and mortality. Whether the water extract of *Lithocarpus litseifolius* (WEL) has therapeutic effects on ALI remains unclear. This study aimed to analyze WEL’s components, establish in vitro cellular inflammation and mouse ALI models, and investigate WEL’s protective effects against LPS-induced ALI. **Methods:** LC-MS analysis identified 42 compounds in WEL and quantified three key ones. In an LPS-induced mouse ALI model, WEL significantly reduced lung injury severity, lung wet-to-dry ratio, pulmonary edema, and levels of NO, ROS, IL-1β, TNF-α, and MPO in lung tissues and bronchial alveolar lavage fluid. Immunohistochemical analysis showed WEL pretreatment inhibited the upregulation of NLRP3, Caspase-1, and GSDMD-NT expression, mitigated tissue oxidative stress and cell pyroptosis, and alleviated ALI severity in mice. Cellular experiments confirmed WEL’s protective effects via anti-inflammatory, antioxidant actions, and inhibiting cell pyroptosis, with phlorizin and trilobatin as potential key active ingredients. **Conclusions:** This research demonstrates sweet tea’s significant protective effects against ALI and its potential to alleviate inflammation by inhibiting pyroptosis, providing a theoretical basis for developing new health-promoting functions of sweet tea.

## 1. Introduction

Acute lung injury (ALI), also known as acute respiratory distress syndrome (ARDS), is a commonly encountered critical condition with high morbidity and mortality rates. It primarily presents as acute hypoxic respiratory failure [1]. Clinical symptoms include refractory hypoxemia, noncardiogenic pulmonary edema, and dyspnea [2].

The pathophysiological process of acute lung injury is highly complex. Previous studies have demonstrated that a severe inflammatory response and oxidative stress can cause significant harm to the body, playing a crucial role in the pathogenesis of acute lung injury and influencing its onset and progression [3]. Research has shown an association between lipopolysaccharide (LPS) and the development of ALI, particularly in triggering the production of reactive oxygen species (ROS). Excessive ROS production can disrupt the body’s redox balance, leading to lung damage, severe inflammatory responses, impaired lung microcirculation, disrupted homeostasis, and pulmonary edema [4]. Excessive ROS accumulation can also induce NLRP3 inflammasome activation, which subsequently activates the Caspase-1/GSDMD pathway, leading to pyroptosis. Pyroptosis is a specialized form of programmed cell death accompanied by an inflammatory response, exhibiting characteristics of both apoptosis and necrosis [5]. It is characterized by the destruction of intact cell membrane structures, cell swelling, release of contents, and inflammatory responses [6]. During pyroptosis, a large quantity of inflammatory factors are released, exacerbating the systemic inflammatory response. Consequently, inhibiting inflammatory responses, oxidative stress, and pyroptosis stands as one of the strategies to combat ALI, with functional foods possessing anti-inflammatory and antioxidant properties showing great potential in its prevention or treatment.

Sweet tea, also known as *Lithocarpus litseifolius* (Hance) Chun, is an evergreen tree in China. The tea brewed from its leaves boasts a mellow and rich flavor. The tea infusion has a limpid hue and a sweet aroma, earning it the folk name ‘sweet tea’ [7]. Sweet tea boasts a long history of consumption in China and has become a common beverage in daily life. It serves as a novel food source with dual properties of both medicine and food [8]. Sweet tea is rich in dihydrochalcones such as trilobatin, phlorizin, and phloretin [8,9]. For instance, phlorizin in sweet tea ameliorates insulin resistance induced by free fatty acids (FFA) by regulating the AMPK/PI3K/AKT signaling pathway [10]. The aqueous extract of sweet tea leaves protects SH-SY5Y cells from hydrogen peroxide-induced damage by activating the Sirt3 signaling pathway [11]. Sweet tea has the potential to prevent ulcerative colitis [12]. This indicates that sweet tea holds enormous potential value in terms of anti-inflammatory and antioxidant properties. However, the comprehensive effects and potential mechanisms of the WEL on acute lung injury have not been reported. Additionally, its effects on the NLRP3/GSDMD pathway remain unclear. The impacts of phloridzin and trilobatin, the principal constituents of WEL, on pyroptosis are still not well-defined.

Therefore, this study has two primary objectives. The first objective is to analyze the components of WEL and determine the contents of three major components (Phlorizin, Phloretin, Trilobatin) with potential nutritional functions. The second objective is to comprehensively utilize animal and cellular experiments to explore the anti-inflammatory, anti-pyroptotic, therapeutic, and protective effects of WEL against acute lung injury, providing a theoretical basis for component research of sweet tea and the development and utilization of anti-inflammatory and antioxidant functional foods.

## 2. Materials and Methods

### 2.1. Materials

Dexamethasone (DEX) and adenosine triphosphate (ATP) were purchased from Aladdin Biochemical Technology Co., Ltd. (Shanghai, China). The Cell Counting Kit-8 (CCK-8) cell proliferation assay kit, one-step TUNEL apoptosis detection kit (with green fluorescence), cell climbing slides, 4′,6-diamidino-2-phenylindole (DAPI) staining solution, fluorescein isothiocyanate (FITC)-labeled goat anti-rabbit IgG (H+L) antibody, cell membrane red fluorescence staining kit, DAF-FM (a fluorescent probe for nitric oxide, NO), and DCFH-DA (a fluorescent probe for reactive oxygen species, ROS) along with its detection kit were all provided by Beyotime Biotechnology Co., Ltd. (Shanghai, China). The antibodies for ASC,F4/80 GSDMD, NLRP3, Caspase-1, and Pro-Caspase-1 were from Affinity Biosciences (Cincinnati, OH, USA), the NO concentration assay kit and the lactate dehydrogenase (LDH) assay kit (microplate method) were from Nanjing Jiancheng Bioengineering Institute (Nanjing, China), the permeabilization buffer, BSA, DAPI staining solution, antifade mounting medium, PBS buffer, anhydrous ethanol, xylene, citrate antigen retrieval solution, hydrogen peroxide, and rabbit serum were from Wuhan Bioruf Biotechnology Co., Ltd., (Wuhan, China) the mouse interleukin-6 (IL-6) ELISA kit, mouse interleukin-1β (IL-1β) ELISA kit, and mouse tumor necrosis factor-α (TNF-α) ELISA kit were from Hangzhou Multi Sciences Biotech Co., Ltd., (Hangzhou, China) lipopolysaccharide (LPS), formic acid and isoflurane were from Sigma (Shanghai, China), chromatographic-grade methanol and chromatographic-grade acetonitrile were from Thermo Fisher Scientific (Shanghai, China) Co., Ltd., and trilobatin (≥98%), phlorizin (≥98%), and phloretin (≥98%) were from Shanghai Standard Biotech Co., Ltd. (Shanghai, China).

### 2.2. WEL Preparation

Sweet tea was collected from Miyi County, Panzhihua City, Sichuan Province, China. It was identified as *Lithocarpus litseifolius* (Hance) Chun, belonging to the genus *Lithocarpus* in the family *Fagaceae*, by Associate Professor Bai Jie from the Botany Department of the College of Life Sciences, Sichuan University. The leaves were dried, powdered, and 100 g of the powder was weighed. Then, 30 times the volume of ultrapure water (sample-to-solvent ratio = 1:30) was added to the powder. The extraction was carried out at 100 °C for 1 h, repeated twice. The filtrates were combined and concentrated to 5–10 mL before being freeze-dried into powder.

Three reference standards of medicinal herbs, trilobatin, phlorizin, and phloretin, were separately weighed at 100 μg. A 1 μg/mL stock solution was prepared by dissolving each reference standard in 70% acetonitrile for chromatography. The stock solution was then diluted to different concentrations for the preparation of external standard calibration curves.

A concentration of 1 μg/mL of WEL was prepared using 70% acetonitrile for chromatography. All the aforementioned samples were filtered using a 0.22 μm PTFE microporous membrane, followed by centrifugation at 12,000 rpm for 5 min. The supernatant was transferred to UPLC sample vials in preparation for analysis.

### 2.3. WEL Untargeted Mass Spectrometry Analysis

The LC-MS acquisition method was IDA mode (information-dependent acquisition mode, i.e., TOF MS-IDA-TOF MS/MS scan mode). The instrument used the ExionLCTMAC liquid phase system and the X500R QTOF mass spectrometry system, and the SCIEX OS 1.6.1 software was used for data analysis.

#### 2.3.1. Liquid-Phase Conditions

Column: Thermo Hypersil Gold 100 × 2.1 (mm), 1.9 microns (25002-102130). Mobile phase A: 0.1% formic acid in water, mobile phase B: acetonitrile. Column temperature: 40 °C. Injection volume: 5 μL. The chromatographic procedure is shown in Table 1.

#### 2.3.2. Mass Spectrometry Conditions

The mass spectrometry conditions required for this experiment are shown in Table 2.

#### 2.3.3. Data Processing

The analysis of untargeted compound data was conducted using the untargeted screening workflow of SCIEX OS software (version 1.7), with the detection sensitivity set to medium peak. The peak areas were statistically analyzed and the dataset was exported. Subsequently, the dataset was imported into SIMCA software (version 13.0, Umea, Sweden) for multivariate statistical analysis.

### 2.4. WEL Targeted Mass Spectrometry Analysis

A volume of 0.8 mL of supernatant was aspirated into a UPLC vial and prepared for instrumental analysis. The experimental setup involved the use of a targeted mass spectrometry instrument in multireaction monitoring (MRM) mode, equipped with a Shimadzu LC30AD liquid chromatography system (Shimadzu Corporation, Kyoto, Japan) and a QTRAP 6500+ mass spectrometry system (SCIEX, Marlborough, MA, USA). The Analyst 1.7.0 software was utilized for both qualitative and quantitative assessments of the samples.

#### 2.4.1. Liquid-Phase Conditions

The column employed was a Thermo Hypersil Gold, with dimensions of 100 × 2.1 mm and a particle size of 1.9 microns (reference number: 25002-102130). The mobile phases consisted of 0.1% formic acid in water (mobile phase A) and acetonitrile (mobile phase B). The column temperature was maintained at 40 °C, and the injection volume was set to 1 μL. The chromatographic procedure is shown in Table 3.

#### 2.4.2. Mass Spectrometry Conditions

An electrospray ionization (ESI) source was employed, utilizing the MRM-IDA-EPI scanning mode for qualitative and quantitative analysis. The electrospray voltage was set at −4500 V; the ion source temperature was maintained at 400 °C; and the auxiliary gas flows for ion source gases GS1/GS2 were both adjusted to 50 L/h.

#### 2.4.3. Data Processing

Standards of trilobatin, phlorizin, and phloretin, encompassing various concentrations, were analyzed under conditions identical to those used for the prepared samples. Standard curves were generated based on the peak areas of these standards across different concentrations. Subsequently, the concentrations of each compound within the samples were calculated.

### 2.5. Animal and Experimental Design

SPF-grade, healthy, four-week-old male BALB/c mice with an average body weight of 16 g were procured from the Hubei Provincial Laboratory Animal Research Center. These mice were meticulously maintained under standardized conditions, including a temperature of 22 ± 2 °C, humidity maintained at 60 ± 5%, and a regulated 12 h light/dark cycle, with ad libitum access to both water and food. Following a one-week period of acclimatization, the experimental protocol commenced. The study was approved by the Ethics Committee of the College of Life Sciences, Sichuan University (Approval No.: SCU230418002). All animal experiments adhered strictly to international norms pertaining to the ethical use and welfare of laboratory animals. In this experiment, mice were first assigned unique identification numbers. Then, they were allocated to different experimental groups based on random numbers generated by a random number generator. The experiment employed a double—blind design, where both the operators and the evaluators were unaware of the specific group details, identifying the mice only by their numbers. At the end of the experiment, an independent researcher carried out the unblinding process.

In order to ensure that the experiment provides sufficient statistical power, balances costs and resources, meets the requirements of experimental design and animal welfare, is supported by previous research and literature, and allows for relatively simple practical operations that facilitate the maintenance of data consistency, a sample size of n = 8 mice per group was adopted. Since measuring certain experimental indicators inevitably involves damaging mouse tissues that cannot be reused (such as in immunohistochemical analysis, tissue reactive oxygen species (ROS) and nitric oxide (NO) detection, etc.), three mice were randomly selected from each group for each indicator measurement. The mouse numbers of the selected mice were generated using a random number table.

The mice were randomly divided into five experimental groups (n = 8), with the specific groupings as follows: the control group, the LPS group, the dexamethasone (10 mg/kg) group, and the experimental (low-dose and high-dose) groups. The specific treatment methods for each group are described as follows:

(1) **Control Group:** The mice in the control group were administered an equal volume of normal saline by gavage once a day. On the seventh day, 2 h after the last administration, the mice were anesthetized with isoflurane. Subsequently, an equal volume of sterile water was injected into the trachea. Six hours after the injection (this injection did not cause any induced injury in this group), the mice were euthanized by cervical dislocation.
(2) **LPS Group:** The treatment method for the mice in the LPS group was the same as that of the control group, with an equal volume of normal saline administered by gavage once a day. On the seventh day, 2 h after the last gavage of normal saline, the mice were anesthetized with isoflurane. Then, LPS (5 mg/kg) was injected into the trachea to induce injury. Six hours after the LPS injection, the mice were euthanized by cervical dislocation.
(3) **Dexamethasone (10 mg/kg) Group:** The mice in the dexamethasone group were administered dexamethasone at a dose of 10 mg/kg by gavage once a day. On the seventh day, 2 h after the last administration of dexamethasone, the mice were anesthetized with isoflurane. Similar to the LPS group, LPS (5 mg/kg) was injected into the trachea to induce injury. Six hours after the LPS injection, the mice were euthanized by cervical dislocation. This group serves as the positive control.
(4) **Experimental (Low-Dose and High-Dose) Groups:** The mice in the low-dose experimental group were administered WEL at a dose of 200 mg/kg by gavage once a day, while the mice in the high-dose experimental group were administered WEL at a dose of 600 mg/kg by gavage once a day. On the seventh day, 2 h after the last administration of WEL, the mice were anesthetized with isoflurane. Then, LPS (5 mg/kg) was injected into the trachea to induce injury. Six hours after the LPS injection, the mice were euthanized by cervical dislocation.

The bronchoalveolar lavage fluid (BALF) and lung tissues were collected and stored at −80 °C for subsequent experiments.

### 2.6. Wet/Dry Lung Weight Ratio

The severity of pulmonary edema in mouse lung tissue was quantified using the wet-to-dry lung weight ratio (W/D ratio). The specific procedure was as follows: Three mice were randomly selected from each experimental group. After euthanizing the selected mice, tissue samples were carefully collected from the upper lobe of their left lungs. During the collection process, the superficial connective tissue of the samples was removed, and excess moisture and blood on the tissues were carefully absorbed using filter paper to ensure the accuracy of the weighing results. Subsequently, the tissues were immediately weighed to obtain the wet weight (wet) of the lungs. Then, the same lung tissues were placed in an oven at 65 °C and dried for 48 h until a constant weight was reached. At this point, they were weighed again to determine the dry weight (dry). Finally, the W/D ratio was calculated for the lung tissue of each mouse, and the average W/D ratio of the three mice in each group was computed. This average value was used as an indicator to assess the severity of pulmonary edema in the corresponding experimental group.

### 2.7. Detection of Inflammatory Factors in BALF

Rinsed the bronchi three times with 2 mL of pre-cooled PBS buffer to collect the BALF. A portion of the BALF was then taken to determine the protein content using the BCA Protein Kit, while the remaining part was centrifuged at 2000 rpm at 4 °C for 10 min. The supernatant was carefully collected and stored at −20 °C for subsequent quantification of the inflammatory factors TNF-α, IL-6, and IL-1β using ELISA kits, adhering strictly to the provided instructions.

### 2.8. ROS and NO Content of Lung Tissue

After rinsing the lung tissues from three randomly selected mice in each experimental group with phosphate-buffered saline (PBS), the tissues were ground into cell suspensions, which were then filtered. Subsequently, reactive oxygen species (ROS) fluorescent probes were added to the filtered suspensions. Following centrifugation at 1400 rpm for 10 min, the cell pellets were collected and washed twice with PBS in preparation for flow cytometry analysis. In addition, tissue homogenates were prepared from the same lung tissues, and the nitric oxide (NO) content in the tissues was measured using a NO concentration assay kit.

### 2.9. Histological Analysis and Immunohistochemistry

Three mice were randomly selected from each experimental group, and lung tissues of similar location and size were harvested from them. These lung tissues then underwent a unified process of 48 h fixation, dehydration, and clearing. Subsequently, the processed lung tissues were embedded in paraffin. After the paraffin hardened, the tissues were sliced into sections with a thickness of 5–8 μm. Next, the sections were subjected to dewaxing and stained with Hematoxylin and Eosin (H&E) for the observation of pathological structures. During the antigen retrieval process, endogenous peroxidase was blocked. After that, the sections were incubated with corresponding primary and secondary antibodies (labeled with horseradish peroxidase, HRP), resulting in brownish-yellow positive staining with diaminobenzidine (DAB) and counterstaining of the nuclei. Finally, the sections were dehydrated and mounted.

### 2.10. Cell Culture

The RAW264.7 cells were purchased from Haixing Biotechnology Co., Ltd. (Suzhou, China). RAW264.7 cells were propagated in high-glucose Dulbecco’s Modified Eagle Medium (DMEM), enriched with 10% FBS. Cultured in bottles, they were maintained at a stable 37 °C temperature and 5% CO_2_ concentration within the incubator. Upon achieving a confluence of approximately 70–80%, the cells underwent passaging for continued culturing. Following this, cells from the logarithmic growth phase were selectively procured for subsequent experimental procedures.

### 2.11. Cell Experiments

#### 2.11.1. Establishment of an LPS-Induced Inflammatory Model in RAW264.7 Cells

RAW264.7 cells in the logarithmic growth phase were seeded into 6-well plates. Once the cell confluence reached 70–80%, the culture medium was aspirated, and the subsequent experiments were conducted as follows:

(1) **Control group**: 2 mL of serum-free medium was added, and the cells were cultured for an additional 11 h. After that, the supernatant was discarded, the cells were washed 2–3 times with PBS, and then 2 mL of serum-free medium was added again for a further 11 h of culture.
(2) **Model group**: 2 mL of serum-free medium was added, and the cells were cultured for 11 h. Subsequently, the supernatant was removed, the cells were washed 2–3 times with PBS, and then treated with 1 μg/mL LPS for 11 h.
(3) **Positive control group**: 2 mL of medium containing 200 μM dexamethasone was added, and the cells were treated for 11 h. Afterward, the supernatant was discarded, the cells were washed 2–3 times with PBS, and then treated with 1 μg/mL LPS for 11 h.
(4) **Experimental groups**: 2 mL of serum-free medium containing WEL at concentrations of 50, 100, and 150 μg/mL, respectively, was added, and the cells were cultured for 11 h. Then, the supernatant was removed, the cells were washed 2–3 times with PBS, and subsequently treated with 1 μg/mL LPS for 11 h.

#### 2.11.2. Establishment of a Cell Pyroptosis Model Induced by the Combined Stimulation of LPS and ATP [13,14]

RAW264.7 cells in the logarithmic growth phase were seeded into 6-well plates. Once the cell confluence reached 70–80%, the culture medium was aspirated, and the subsequent experiments were carried out as follows:

(1) **Control group**: 2 mL of serum-free medium was added, and the cells were cultured for an additional 11 h. After that, the supernatant was discarded, the cells were washed 2–3 times with PBS, and then 2 mL of serum-free medium was added again for a further 11 h of culture. Subsequently, the supernatant was removed, the cells were washed 2–3 times with PBS, and 2 mL of serum-free medium was added for a 30 min treatment.
(2) **Model group**: 2 mL of serum-free medium was added, and the cells were cultured for 11 h. Then, the supernatant was discarded, the cells were washed 2–3 times with PBS, and treated with 1 μg/mL LPS for 11 h. Afterward, the supernatant was removed, the cells were washed 2–3 times with PBS, and 2 mL of serum-free medium containing 5 mM ATP was added for a 30 min treatment.
(3) **Positive control group**: 2 mL of medium containing 200 μM dexamethasone was added, and the cells were treated for 11 h. Then, the supernatant was discarded, the cells were washed 2–3 times with PBS, and treated with 1 μg/mL LPS for 11 h. Subsequently, the supernatant was removed, the cells were washed 2–3 times with PBS, and 2 mL of serum-free medium containing 5 mM ATP was added for a 30 min treatment.
(4) **Experimental groups**: 2 mL of serum-free medium containing WEL at concentrations of 50, 100, and 150 μg/mL, respectively, was added, and the cells were cultured for 11 h. Then, the supernatant was discarded, the cells were washed 2–3 times with PBS, and treated with 1 μg/mL LPS for 11 h. Afterward, the supernatant was removed, the cells were washed 2–3 times with PBS, and 2 mL of serum-free medium containing 5 mM ATP was added for a 30 min treatment.

### 2.12. Scanning Electron Microscopy to Observe Cell Morphology

The coverslips were placed into a 6-well plate. After treating RAW264.7 cells with LPS and ATP, the culture medium was removed, and the cell morphology was fixed using a special fixative for scanning electron microscopy. The coverslips were then washed twice with UP water for 5 min each time, followed by dehydration in a series of gradient alcohols, with each gradient lasting 10 min. The slides were gently affixed to conductive adhesive, subjected to ion sputter coating, and finally positioned appropriately under the microscope for observation at the desired magnification.

### 2.13. Cell NO and ROS Contents

RAW264.7 cells in the logarithmic growth phase were harvested and seeded into 6-well plates at a density of 2 × 10^5^ cells/mL. After treatment according to Section 2.11.1 or Section 2.11.2, the cells were washed twice with phosphate-buffered saline (PBS). Subsequently, 500 μL of 10 μM DAF-FM (for nitric oxide, NO) or DCFH-DA (for reactive oxygen species, ROS) probe was added to the control group, model group, and experimental group, respectively. The cells were then incubated in a cell culture incubator at 37 °C in the dark for 30 min before being transferred to flow cytometry tubes for cell flow cytometric analysis. For the measurement of extracellular NO content, after drug treatment of the cells, the cell culture supernatant was collected and assayed using an NO assay kit, with the operational procedures carried out in accordance with the kit’s instructions.

### 2.14. TUNEL Staining and Immunofluorescence Staining

Cells were cultured on coverslips. After treatment with LPS and ATP, a TUNEL staining kit was used to assess DNA damage in Raw264.7 cells. Following the kit instructions, the level of TUNEL-positive cells was detected, and cell nuclei were stained with DAPI for 5 min before observation under a fluorescence microscope. In a separate experiment, cells were seeded in a 24-well plate containing cell climbing slices. After treatment, the membrane localization of the GSDMD protein was examined. The GSDMD protein was labeled with an antibody containing a specific fluorophore, while cell nuclei were stained with DAPI, and cell membranes were stained with DIL as a reference to determine the specific location of the target protein.

### 2.15. Statistical Analysis

When an experiment has multiple treatment groups (such as drug treatment groups with different concentrations), One-way ANOVA can be used to make a preliminary determination of whether there are overall significant differences in the means among these groups. Therefore, in this experiment, One-way ANOVA is uniformly adopted for statistical analysis. The Prism 8.0 software was used for data analysis and graphing. All experimental results are presented as Mean ± SD (mean ± standard deviation) with n = 3. A significance level of *p* < 0.05 was considered.

## 3. Results

### 3.1. WEL Components Analysis and Principal Components Qualitative Quantification

**Figure 1:**
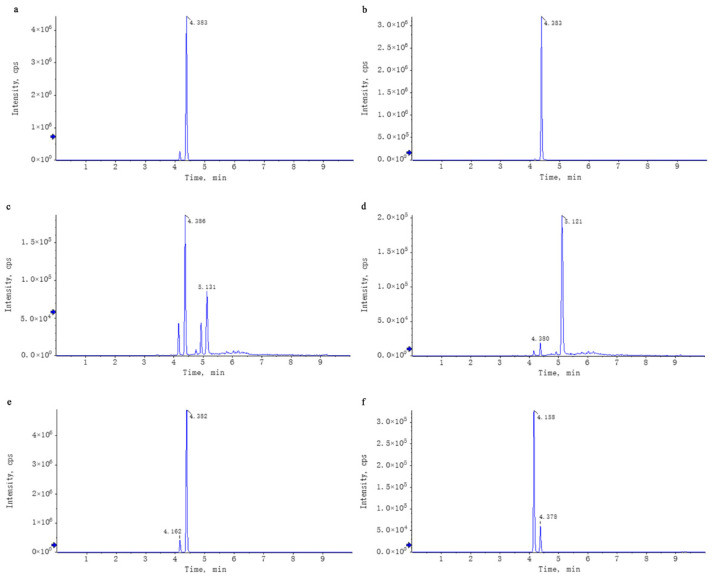
Comparison of the XIC of the standards and the corresponding compounds in WEL. Note: (**a**,**b**) trilobatin, (**a**): trilobatin in WEL; (**b**): standards (**c**,**d**) phloretin, (**c**): phloretin in WEL; (**d**): standards (**e**,**f**) phlorizin, (**e**): phlorizin in WEL; (**f**): standards.

**Figure 2:**
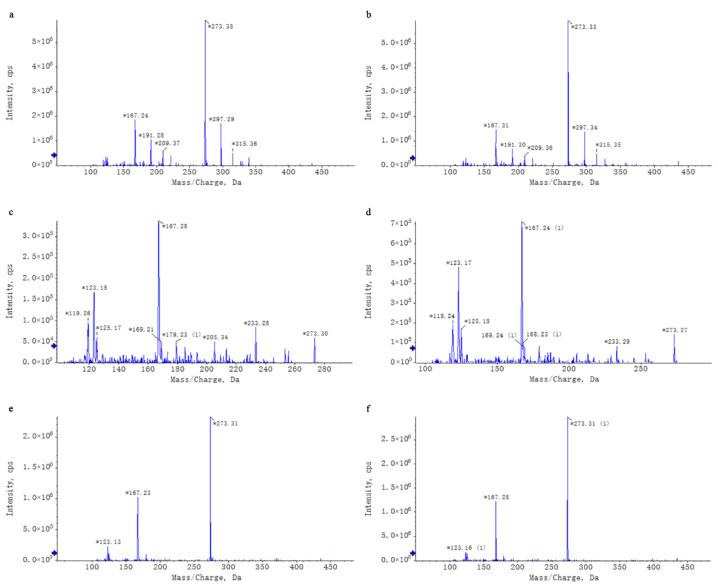
Chromatographic Comparison of Standard Compounds and Their Corresponding Counterparts in WEL. Note: (**a**,**b**) trilobatin, (**a**): trilobatin in WEL; (**b**): standards (**c**,**d**) phloretin, (**c**): phloretin in WEL; (**d**): standards (**e**,**f**) phlorizin, (**e**): phlorizin in WEL; (**f**): standards. * Key peaks in the compound’s chromatogram.

Untargeted mass spectrometry analysis identified 42 compounds in WEL, such as phlorizin (Table 4). The mass spectrometry database, a non-public one, was provided by the Mass Spectrometry Platform of the College of Life Sciences at Sichuan University. Due to the limited data in the untargeted mass spectrometry database and literature indications that the aqueous extract of *Lithocarpus litseifolius* contains major compounds like trilobatin, phlorizin, and phloretin [8], targeted mass spectrometry comparisons were conducted between standard compounds and WEL to qualitatively and quantitatively analyze these three compounds in WEL. As shown in Table 5, the content of trilobatin in WEL is 465.5 ng/μg, the content of phloretin is 1.31 ng/μg, and the content of phlorizin is 49.29 ng/μg. The comparison charts of extracted ion chromatograms between the standard compounds and their corresponding counterparts in WEL are shown in Figure 1. The comparative mass spectra of the three main components between the standard compounds and WEL are shown in Figure 2. Therefore, WEL is rich in trilobatin and phlorizin, two nutrients beneficial to human health. Subsequent cellular experiments will verify whether these two main components inhibit inflammatory responses through pyroptosis.

### 3.2. WEL Alleviates LPS-Induced Acute Lung Injury in Mice

**Figure 3:**
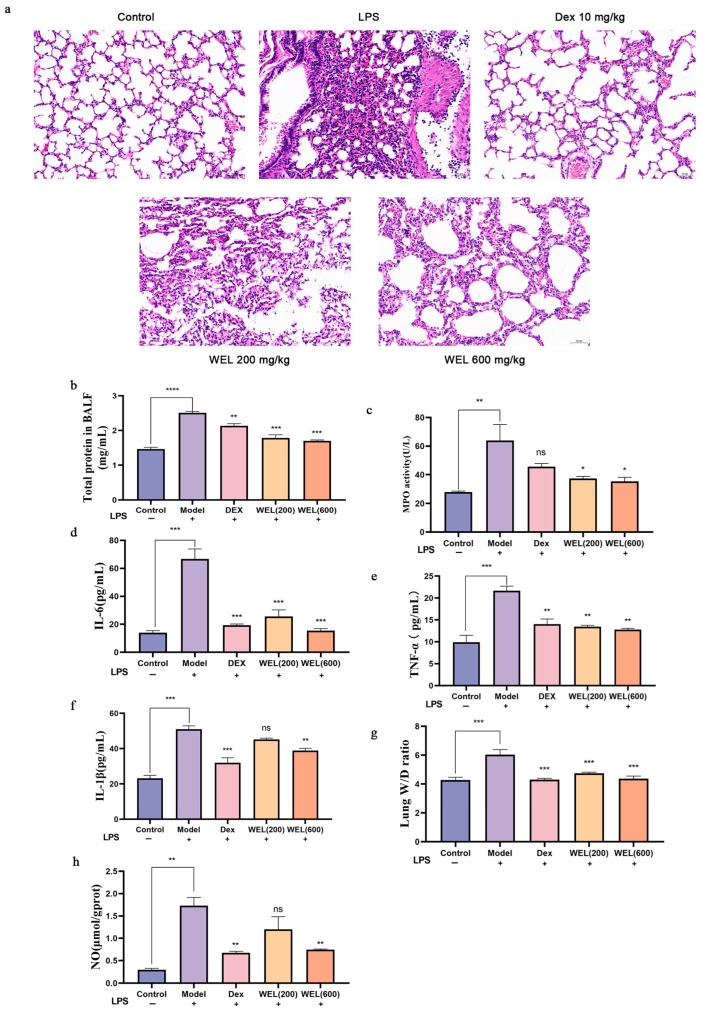
LPS-induced acute lung injury phenotype in mice (**a**) WEL pretreatment alleviates LPS-induced lung injury in mice (**b**) Total protein contents in BALF; (**c**) MPO in BALF; (**d**–**f**) IL-6, TNF-α, IL-1β in BALF; (**g**) Individual and mean values of W/D ratio measured from the ratio of mouse wet lung weight to dry lung weight; (**h**) NO content in mouse lungs. The experimental results are expressed as mean ± standard deviation (Mean ± SD), with n = 3. ns: no significant difference; * *p* < 0.05, ** *p* < 0.01, *** *p* < 0.001, **** *p* < 0.0001, compared with the Model group.

As depicted in Figure 3a, the control group’s lung tissue exhibited an overall normal structure, characterized by clear and intact alveolar architecture. Conversely, the LPS-treated group displayed marked abnormalities in lung tissue, encompassing alveolar atrophy, detachment and swelling of epithelial cells, thickening and slight consolidation of alveolar walls, significant congestion in lung interstitium, and prominent inflammatory cell infiltration. The positive control Dex group and the WEL pre-treatment group had mitigated these LPS-induced effects, preserving lung morphology and ameliorating alveolar atrophy, consolidation and inflammatory cell infiltration. These findings had visually underscored WEL’s potent inhibitory effect on LPS-mediated lung injury and its protective role.

Furthermore, Figure 3b illustrates that BALF total protein levels, indicative of lung inflammation severity, were significantly elevated in LPS-exposed mice compared to controls, accompanied by a surge in IL-1β, IL-6, and TNF-α cytokines. Notably, WEL pre-treatment had markedly reduced BALF protein content and the levels of these inflammatory markers (Figure 3d–f), suggesting reversal of LPS-induced neutrophil, macrophage, and cytokine increases.

Regarding pulmonary edema, the lung wet-to-dry weight ratio revealed that LPS induction had exacerbated edema in mice compared to the control group, whereas WEL pre-treatment had attenuated this effect, demonstrating lung protection (Figure 3g). Additionally, MPO activity, a neutrophil-specific biomarker, had been downregulated by WEL in LPS-challenged mouse lung tissue (Figure 3c), suggesting reduced neutrophil accumulation.

INOS, a catalyst for NO overproduction and marker of M1 macrophage polarization, had been upregulated by LPS, leading to heightened NO levels in lung tissue. However, WEL pre-treatment had significantly mitigated this LPS-induced NO production (Figure 3h), highlighting its modulatory effect on macrophage phenotype and function.

### 3.3. WEL Inhibits the Generation of Reactive Oxygen Species in the Lungs of Mice

**Figure 4:**
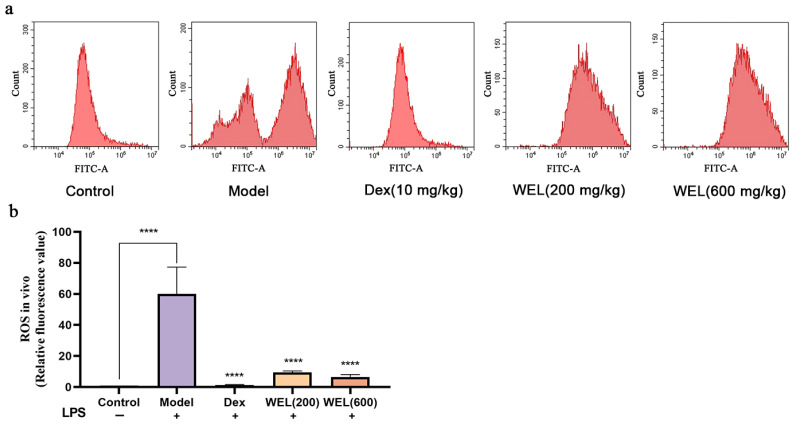
WEL inhibits the generation of reactive oxygen species in the lungs of mice. (**a**) Flow cytometry to detect ROS levels in mouse lungs; (**b**) plot of ROS in mouse lungs. The experimental results are expressed as mean ± standard deviation (Mean ± SD), with n = 3. **** *p* < 0.0001, compared with the Model group.

Flow cytometry results (Figure 4a,b) showed that WEL pretreatment reduces the substantial ROS induced by LPS, thus lowering oxidative stress levels in mouse lung tissue and ultimately providing a protective effect on the lungs.

### 3.4. WEL Inhibits Pyroptosis Through the NLRP3/GSDMD Pathway

**Figure 5:**
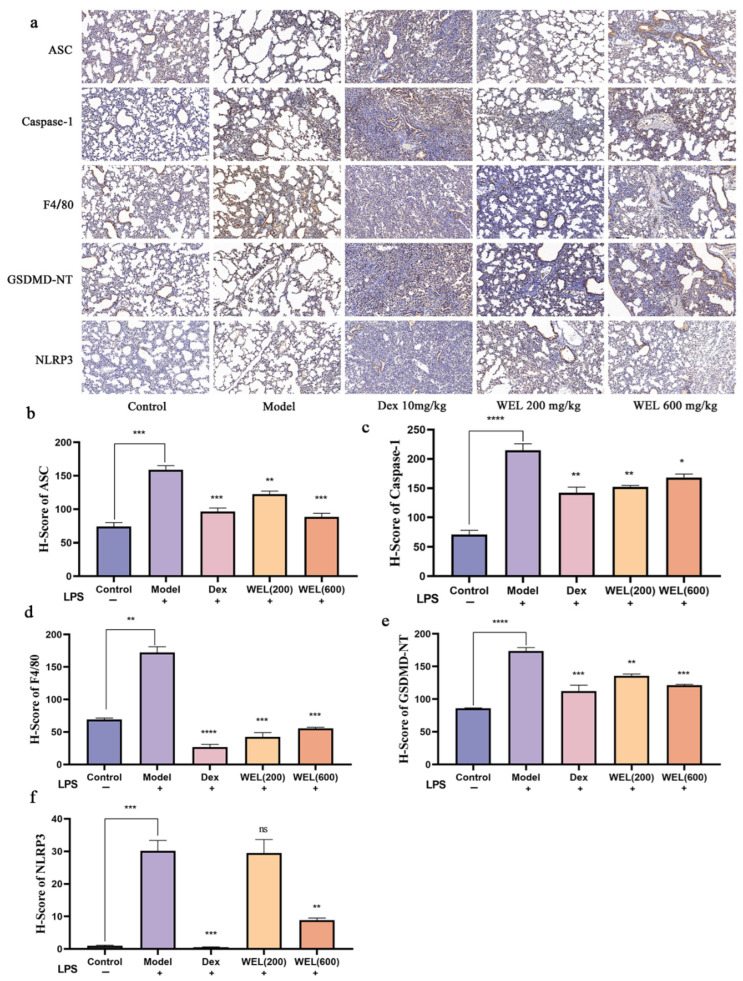
Immunohistochemical analysis. (**a**) Immunohistochemical analysis of ASC, Caspase-1, F4/80, GSDMD-NT, NLRP3; (**b**) H-Score of ASC; (**c**) H-Score of Caspase-1; (**d**) H-Score of F4/80; (**e**) H-Score of GSDMD-NT; (**f**) H-Score of NLRP3. The experimental results are expressed as mean ± standard deviation (Mean ± SD), with n = 3. ns: no significant difference; * *p* < 0.05, ** *p* < 0.01, *** *p* < 0.001, **** *p* < 0.0001, compared with the Model group.

As a classic biomarker of oxidative stress, ROS can induce the activation of NLRP3 inflammasome, which in turn activates the Caspase-1/GSDMD pathway, resulting in pyroptosis. F4/80 is a cell surface glycoprotein that is widely present on the surface of mature macrophages and is a macrophage marker. The immunohistochemical analysis results indicated a significant increase in the expression of NLRP3, ASC, GSDMD-NT, F4/80, and Caspase-1 with LPS induction. However, WEL pretreatment effectively reversed the expression of these proteins, thereby inhibiting pyroptosis (Figure 5a–f).

### 3.5. WEL Inhibits LPS-Induced RAW264.7 Inflammatory Response

**Figure 6:**
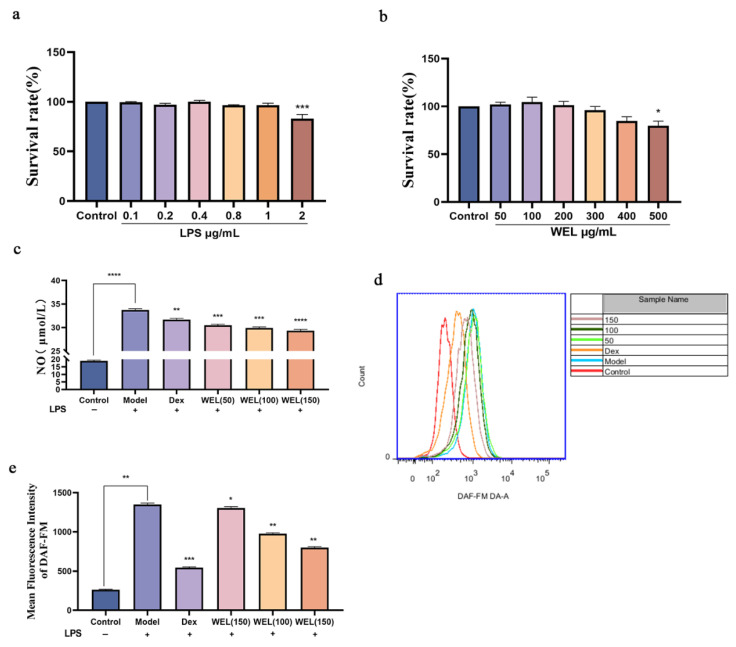
(**a**) LPS on cell viability (**b**) The effect of WEL on cell viability (**c**) Extracellular NO contents of RAW264.7 (**d**) The contents of NO were detected by flow cytometry; (**e**) The quantitative figure of NO. The experimental results are expressed as mean ± standard deviation (Mean ± SD), with n = 3. * *p* < 0.05, ** *p* < 0.01, *** *p* < 0.001, **** *p* < 0.0001, compared with the Model group.

The cytotoxic effect of WEL on cells was evaluated using the CCK-8 assay. After treating RAW264.7 cells with different concentrations of LPS for 11 h, it was found (as shown in Figure 6a) that there was no significant difference in cell viability compared to the Control group (untreated group) when LPS concentrations ranged from 0.1 to 1 μg/mL. Therefore, 1 μg/mL of LPS was selected as the modeling concentration. Subsequently, the cells were treated with WEL, and the results (Figure 6b) indicated that WEL exhibited almost no toxicity at concentrations below 400 μg/mL. Furthermore, Figure 6e shows that after treatment with 1 μg/mL LPS, the extracellular NO concentration significantly increased. However, in RAW264.7 cells pre-treated with WEL for 11 h, the extracellular NO concentration significantly decreased in a dose-dependent manner. Therefore, 50, 100, and 150 μg/mL were chosen as the treatment concentrations for WEL.

Subsequent to pretreatment with varying concentrations of WEL for 11 h, RAW264.7 cells were exposed to LPS for an additional 11 h. The extracellular NO concentration was measured using a test kit. The DAF-FM fluorescent probe was employed to label intracellular NO, and the intracellular NO content was analyzed via flow cytometry. Analysis presented in Figure 6c,d indicates a marked elevation in intracellular NO content following LPS treatment. In contrast, a notable decrease in NO content was observed after pretreatment with Dex and WEL, exhibiting a dose-dependent effect. Compared to the model group, a pronounced shift in peak value was discernible post-pretreatment with various WEL concentrations. Quantitative assessment revealed that WEL pretreatment effectively inhibited the LPS-induced inflammatory response in cells, resulting in a significant decline in NO accumulation.

### 3.6. WEL Inhibits LPS-Induced Oxidative Stress in RAW264.7 Cells

**Figure 7:**
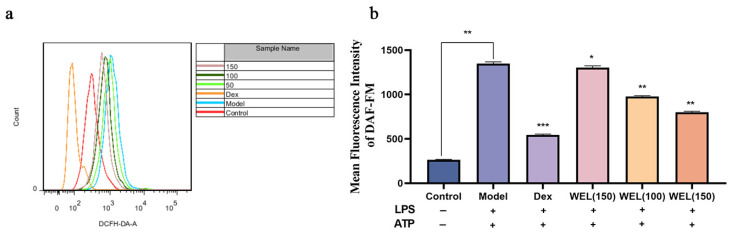
WEL inhibits LPS-induced oxidative stress (**a**) The quantitative figure of ROS; (**b**) The quantitative results of ROS levels in cells. The experimental results are expressed as mean ± standard deviation (Mean ± SD), with n = 3. * *p* < 0.05, ** *p* < 0.01, *** *p* < 0.001, compared with the Model group.

A fluorescent probe was used to label ROS. Employing flow cytometry for the quantification of fluorescently labeled cells (Figure 7a,b), it became evident that WEL pretreatment reversed the elevated expression of ROS observed in the model group. This decrease exhibited a dose-dependent trend, suggesting that WEL pretreatment effectively mitigated ROS production, thereby alleviating oxidative stress within the cells.

### 3.7. WEL Inhibits the Expression of GSDMD and Exerts Anti-Pyroptosis In Vitro

**Figure 8:**
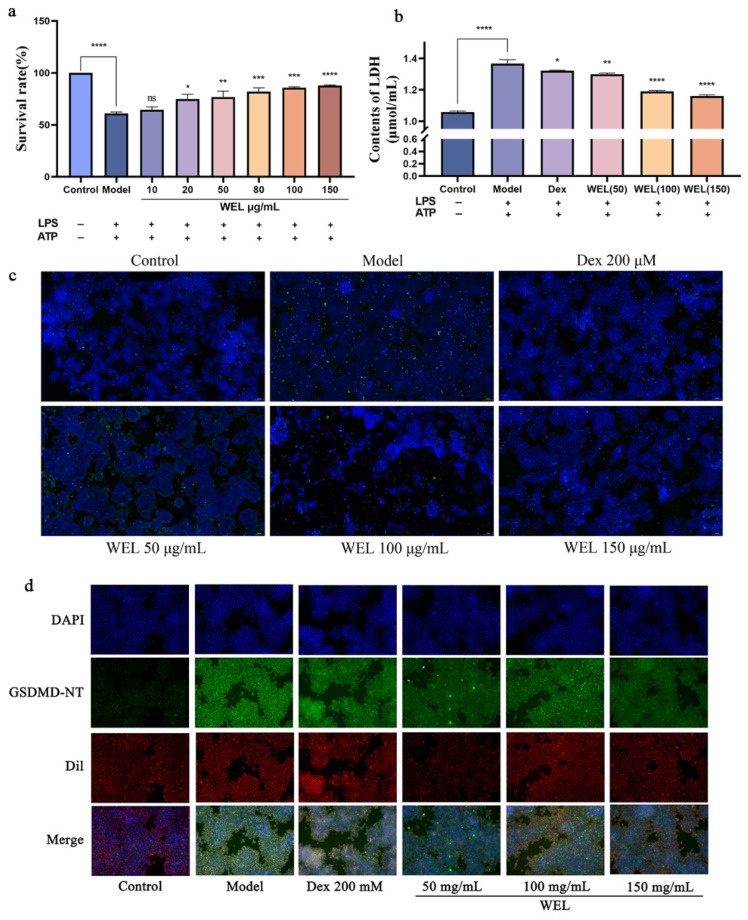
WEL pretreatment can alleviate pyroptosis. (**a**) Cell viability after pyroptosis; (**b**) Contents of LDH; (**c**) WEL pretreatment reduces TUNEL positivity; (**d**) Immunofluorescence staining of GSDMD-NT. The experimental results are expressed as mean ± standard deviation (Mean ± SD), with n = 3. ns: no significant difference; * *p* < 0.05, ** *p* < 0.01, *** *p* < 0.001, **** *p* < 0.0001, compared with the Model group.

To evaluate the pyroptosis model, cell viability and lactate dehydrogenase (LDH) content were assessed using the CCK-8 assay. As pyroptosis involves the formation of membrane pores leading to the release of cellular contents, we measured LDH levels in the supernatant. Our findings indicated that the combined induction of LPS and ATP significantly decreased cell viability (Figure 8a) and caused an increase in LDH leakage, reflected by elevated LDH content in the medium (Figure 8b). However, WEL pretreatment mitigated this effect by reducing extracellular LDH content and improving cell viability, suggesting its protective role.

During pyroptosis, chromosomal DNA fragmentation generates numerous sticky 3′-OH ends, which were detected using the TUNEL kit to quantify DNA damage. The model group exhibited an increase in TUNEL-positive cells after LPS and ATP induction, but WEL pretreatment significantly reversed this trend (Figure 8c), indicating its ability to protect against DNA damage associated with pyroptosis.

Oxidative stress triggers the NLRP3/GSDMD pathway, where GSDMD cleavage releases N-terminal fragments crucial for pyroptosis. ROS activates NLRP3 inflammasomes, subsequently activating the Caspase-1/GSDMD pathway to induce pyroptosis. Immunofluorescence experiments (Figure 8d) revealed that WEL reduced GSDMD protein expression.

### 3.8. The Main Components of WEL, Trilobatin and Phlorizin Monomers, Exert Anti-Pyroptosis Effects In Vitro

**Figure 9:**
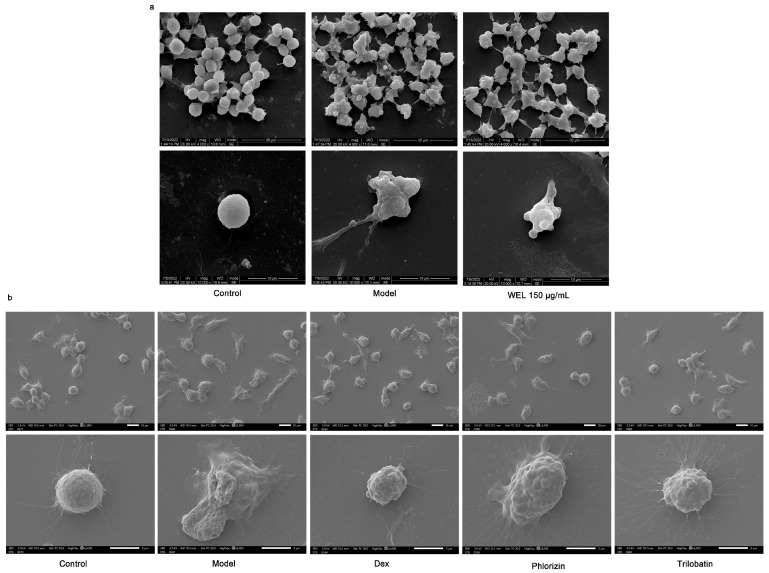
WEL and the monomers trilobatin and phlorizin can inhibit pyroptosis. (**a**,**b**) Scanning electron microscopy to observe cell morphology at the time of pyroptosis.

Scanning electron microscopy images (Figure 9a) demonstrated morphological changes in RAW264.7 cells upon LPS and ATP induction. Compared to the control group’s rounded cells, induced cells became swollen, deformed, and exhibited membrane perforations with efflux of cellular contents. In contrast, WEL pretreatment improved cell morphology, with most cells retaining a rounded shape, reduced swelling, and fewer membrane perforations, suggesting WEL’s protective effect against pyroptosis in RAW264.7 cells. To investigate the suppressive impact of trilobatin and phlorizin on pyroptosis, we utilized scanning electron microscopy to observe the count of pyroptotic cells, elicited by LPS/ATP, subsequent to pre-exposure to these compounds. Our observations revealed a reduction in the number of pyroptotic cells, in contrast to the model group, accompanied by a more preserved cellular morphology (Figure 9b).

## 4. Discussion

ALI, frequently evolving into ARDS, poses significant challenges to global healthcare due to its high morbidity and mortality rates [15]. For example, protein-rich pulmonary edema characteristic of acute lung injury can cause hypoxemia, dyspnea, increased work of breathing, and eventually respiratory failure [16]. The body’s inflammatory response is its physiological reaction to various pathological injuries and stimuli. It is commonly believed that the primary pathogenesis of acute lung injury may stem from an inflammatory response that is challenging to control [17,18]. Uncontrolled oxidative stress also plays a key role in the development of acute lung injury, producing large amounts of reactive oxygen species when the body is stimulated by certain risk factors [19]. Excess ROS exceeds the body’s ability to clear and damages cells and lung tissue, leading to pulmonary edema [20]. Pyroptosis serves as a significant mechanism in acute lung injury. A plethora of pyroptotic phenomena have been observed in the lungs of mice afflicted by acute lung injury [21,22]. When pyroptosis occurs, a large number of inflammatory factors are produced, which induce cytokine storms, aggravate the acute lung injury, and increase the severe disease rate and the difficulty of curing acute lung injury. Hence, the onset of acute lung injury is intricately linked to pyroptosis [23].

LPS is endotoxins mainly distributed on the outer wall of the cell wall of Gram-negative bacteria [24], and LPS activates NADPH oxidase, resulting in a large accumulation of ROS [25]. The pathological characteristics of acute lung injury in mice induced by LPS are similar to those in humans and can trigger excessive secretion of inflammatory mediators, including TNF-α, IL-6, and IL-1β, which are hallmarks of acute lung injury [26]. After LPS induces cells, a large amount of ROS is produced, and excess ROS activates NLRP3 inflammasomes, which in turn activate Caspase-1 to participate in the activation of GSDMD, thereby inducing pyroptosis. ATP induces the assembly of NLRP3 by increasing the concentration of cellular calcium ions [13]. Therefore, LPS and ATP are often used to establish an in vitro cell pyroptosis model [14].

Various western medications with anti-ALI effects such as dexamethasone, methylprednisolone, prednisone, and atorvastatin are widely utilized in the clinical management of ALI. However, these medications can trigger various adverse reactions, including coagulation disorders, gastric ulcers, and osteoporosis, significantly constraining their utilization [27]. Therefore, it is of great importance to seek functional foods for disease prevention or adjuvant therapy. In natural plant products, there are various active small molecules that can alleviate acute lung injury, such as polyphenolic flavonoids [28]. Polyphenolic compounds play an important role in the prevention and treatment of various human diseases. Studies have been conducted on aspects such as cancer, cardiovascular and cerebrovascular diseases, and diabetes [29,30,31]. Polyphenols have a variety of pharmacological effects on oxidative stress and anti-inflammatory processes, which are pathological effects related to the process of acute lung injury. This makes plant polyphenols widely considered in the treatment of inflammation-related diseases [32,33].

Studies have shown that the pathogenesis of acute lung injury may be related to inflammation, oxidative stress, and pyroptosis [34,35,36]. Therefore, edible polyphenols, as well as tea drinks, are effective in preventing lung injury. In our experiment, we simulated people’s usual habit of drinking sweet tea by using the water extract of sweet tea, aiming to be closer to the way sweet tea is consumed in daily life. Given that WEL is a food material and relatively safe [37], the doses for animal experiments were referenced from previously reported doses in the literature [12,38,39]. We established an LPS-induced mouse model of ALI. The protein content and inflammatory cytokine levels in bronchoalveolar lavage fluid (BALF), as well as the lung wet-to-dry weight ratio, are important indicators for assessing acute lung injury. Our experimental results demonstrated that WEL pretreatment could reduce the protein concentration and inflammatory cytokine levels in BALF, as well as alleviate pulmonary edema. Histopathological sections revealed that WEL pretreatment reversed the pulmonary tissue abnormalities and alveolar wall thickening induced by LPS, along with reducing inflammatory cell infiltration, indicating a protective effect of WEL on lung tissue. Furthermore, WEL pretreatment decreased the ROS content in lung tissue. Immunohistochemical analysis showed that compared to the control group, the LPS-treated group exhibited significantly increased expression of F4/80, NLRP3, Caspase-1, ASC, and GSDMD-NT proteins, whereas WEL pretreatment reversed this trend. These findings suggest that WEL alleviates acute lung injury in mice through anti-inflammatory and antioxidant mechanisms, thereby inhibiting pyroptosis. Subsequently, we established the RAW264.7 cell inflammation model using LPS. The expression of various inflammatory indices, such as NO, inflammatory factors, and inflammation-related proteins, increased significantly in cells after LPS treatment. However, after WEL pretreatment, the production of NO and the expression of inflammation-related proteins were significantly inhibited. The GSDMD-NT protein disrupts cell membrane integrity, triggering pyroptosis and the release of lactate dehydrogenase along with numerous inflammatory factors [40,41]. This further strengthens the inflammatory response. The experimental results showed that WEL pretreatment could protect the integrity of cell membranes and reduce the release of ROS and LDH.

Next, we further explored the in-depth mechanism of WEL in inhibiting pyroptosis, which is an important antioxidant pathway that has been shown to play a crucial role in LPS-induced ROS accumulation [42]. Studies have shown that ROS activates the NLRP3 inflammasome and triggers downstream pathways to induce pyroptosis [43,44,45,46]. Pyroptosis can further aggravate the inflammatory response, so inhibiting pyroptosis is also an important approach to prevent acute lung injury. When pyroptosis occurs, DNA in the cell can be damaged and broken [47]. We induced the pyroptosis model using LPS and ATP and measured the extent of DNA damage. It was found that the pretreatment groups with WEL could significantly inhibit the expression of pyroptosis-related proteins and protect DNA from damage. These findings suggest that WEL has certain application value in preventing acute lung injury. Sweet tea is a functional food rich in dietary polyphenols.

## 5. Conclusions

In summary, we identified 42 components in the water extract of *Lithocarpus litseifolius* (Hance) Chun using mass spectrometry and quantified the contents of its three main components: phloridzin, phloretin, and trilobatin. The content of trilobatin in WEL is 465.5 ng/μg, the content of phloretin is 1.31 ng/μg, and the content of phlorizin is 49.29 ng/μg. WEL prevents the cascading amplification of inflammatory responses and alleviates acute lung injury in mice through its anti-inflammatory, antioxidant, and pyroptosis-inhibiting effects. We also observed that WEL exerts anti-inflammatory effects on LPS-induced RAW264.7 cells and provides protective effects against pyroptosis induced by the combination of LPS and ATP. Furthermore, we found that the main components of WEL, trilobatin and phloridzin, can inhibit pyroptosis. The research results indicate that WEL pretreatment can mitigate LPS-induced acute lung injury in mice, with trilobatin and phloridzin playing crucial roles. This suggests that sweet tea exhibits significant anti-inflammatory and antioxidant effects and is a healthy tea beverage. The principal components, trilobatin and phloridzin, are suitable for development into anti-inflammatory drugs or health supplements.

## Figures and Tables

**Table 1:** LC program of Section 2.3.1.

Time	Flow Rate (mL/min)	Mobile Phase B
0	0.4	5
1	0.4	5
6	0.4	95
8	0.4	95
8.1	0.4	2
10	0.4	2

**Table 2:** MS condition.

Name	Condition
Acquisition Time	10 min
Nebulizing Gas	50 psi
Drying Gas	50 psi
Curtain Gas	35 psi
Ion Source Temperature	500 °C
Collision Gas	7 psi
Scan Mode	Negative
Ion Source Voltage	−4500
Scan Range (MS)	50–1000 Da

**Table 3:** LC program of Section 2.4.1.

Time	Flow Rate (mL/min)	Mobile Phase B
0	0.4	5
0.5	0.4	5
6	0.4	95
8	0.4	95
8.1	0.4	5
10	0.4	5

**Table 4:** LC-MS data of the compounds in WEL detected in negative mode.

No.	RT (min)	LC-MS [M-H]^−^	Molecular Formula	Tentative Identification
1	0.73	192.06	C_7_H_12_O_6_	Quinic acid
2	0.92	174.05	C_7_H_10_O_5_	Shikimic acid
3	3.4	435.12	C_12_H_24_O_10_	Phlorizin
4	4.24	154.06	C_8_H_10_O_3_	Hydroxytyrosol
5	4.85	354.095	C_16_H_18_O_9_	Neochlorogenic acid
6	5.02	312.04	C_13_H_12_O_9_	Caftaric acid
7	5.22	449.10	C_21_H_21_O_11_	Asterin
8	5.5	290.07	C_15_H_14_O_6_	(+)-Catechin
9	5.55	354.09	C_16_H_18_O_9_	Chlorogenic acid
10	5.79	354.09	C_16_H_18_O_9_	Cryptochlorogenic acid
11	5.83	168.04	C_8_H_8_O_4_	Vanillic Acid
12	5.84	180.04	C_9_H_8_O_4_	Caffeic acid
13	6.26	290.07	C_15_H_14_O_6_	(-)-Epicatechin
14	6.52	516.12	C_25_H_24_O_12_	1,3-Dicaffeoylquinic acid
15	6.74	786.70	C_35_H_46_O_20_	Echinacoside
16	6.83	152.04	C_8_H_8_O_3_	Vanillin
17	6.9	164.16	C_9_H_8_O_3_	p-Coumaric acid
18	7.22	640.58	C_29_H_36_O_16_	Plantamajoside
19	7.27	610.15	C_27_H_30_O_16_	Rutin
20	7.33	302.00	C_14_H_6_O_8_	Ellagic acid
21	7.38	194.18	C_10_H_10_O_4_	Ferulic acid
22	7.45	464.09	C_21_H_20_O_12_	Isoquercitrin
23	7.54	304.05	C_15_H_12_O_7_	Taxifolin
24	7.54	624.20	C_29_H_36_O_15_	Verbascoside
25	7.73	594.52	C_27_H_30_O_15_	Nicotiflorin
26	7.9	516.12	C_25_H_24_O_12_	Isochlorogenic acid A
27	7.9	516.12	C_25_H_24_O_12_	1,5-Dicaffeoylquinic acid
28	7.92	516.12	C_25_H_24_O_12_	Isochlorogenic acid B
29	7.94	448.10	C_21_H_20_O_11_	Astragalin
30	8.31	516.12	C_25_H_24_O_12_	Isochlorogenic acid C
31	8.51	286.23	C_15_H_10_O_6_	Fisetin
32	8.63	474.07	C_22_H_18_O_12_	Chicoric acid
33	8.72	302.04	C_15_H_10_O_7_	Tricetin
34	9.85	288.25	C_15_H_12_O_6_	Fustin
35	9.85	288.06	C_15_H_12_O_6_	Eriodictyol
36	10	286.04	C_15_H_10_O_6_	Luteolin
37	10.02	302.04	C_15_H_10_O_7_	Quercetin
38	10.39	272.06	C_15_H_12_O_5_	Naringenin
39	10.42	148.05	C_15_H_10_O_5_	Apigenin
40	10.47	286.04	C_15_H_10_O_6_	Kaempferol
41	10.48	330.29	C_17_H_14_O_7_	Tricin
42	10.9	148.05	C_9_H_8_O_2_	Cinnamic acid

**Table 5:** The quantitative analysis parameters and contents in WEL.

Compounds	Linear Equation	R^2^	Content in WEL (ng/μg)
trilobatin	y = 14,022.001435 x + −5658.83325	0.99872	465.5
phloretin	y = 7.84933 × 10^4^ x + 24,591.13457	0.99696	1.31
phlorizin	y = 10,660.73682 x + −5404.51522	0.99715	49.29

## Data Availability

The data presented in this study are available on request from the corresponding author due to ethical/privacy restrictions.
